# Preparation of anthracene-based tetraperimidine hexafluorophosphate and selective recognition of chromium(III) ions

**DOI:** 10.3762/bjoc.15.278

**Published:** 2019-11-25

**Authors:** Qing-Xiang Liu, Feng Yang, Zhi-Xiang Zhao, Shao-Cong Yu, Yue Ding

**Affiliations:** 1 Tianjin Key Laboratory of Structure and Performance for Functional Molecules, MOE Key Laboratory of Inorganic-Organic Hybrid Functional Material Chemistry, College of Chemistry, Tianjin Normal University, Tianjin 300387, P. R. China

**Keywords:** chemosensor, chromium(III) ion, tetraperimidine

## Abstract

A novel anthracene-based tetraperimidine hexafluorophosphate **3** was prepared, and its structure was determined through X-ray analysis, HRMS as well as ^1^H and ^13^C NMR spectroscopy. In the cationic moiety of **3**, two (*N*-ethylperimidinyl–C_2_H_4_)_2_NCH_2_– arms were attached to the 9- and 10-positions of anthracene. In addition, compound **3** was used as a chemosensor to research the ability to recognize Cr^3+^ through fluorescence and UV titrations, HRMS, as well as ^1^H NMR and IR spectroscopy. The results indicate that **3** is an effective chemosensor for Cr^3+^.

## Introduction

Fluorescent chemosensors are an attractive and efficient tool for the detection of metal ions in environmental and biological science because of their high sensitivity, selectivity, and simple usage [1–8]. Among metal ions, the detection of Cr^3+^ ions occupies an important position. Chromium(III) is an essential microelement for humans and animals, and it plays an important role in glucose and lipid metabolism in the body [9–10]. The deficiency of chromium(III) in the human body leads to various diseases, such as diabetes as well as autoimmune and cardiovascular diseases [11]. On the other hand, the excessive incorporation of chromium(III) is toxic to humans, and can cause cancer through the oxidation of DNA and some proteins [12–14]. Therefore, the detection of chromium(III) has a vital practical significance for human health monitoring.

In recent years, some fluorescent chemosensors for the detection of chromium(III) have been developed [15–23]. Generally, chemosensors with fluorescence enhancement are more efficient than fluorescence turn-off chemosensors [24–29] because the paramagnetic nature of chromium(III) can cause fluorescence quenching of the fluorophore via the enhancement of spin–orbit coupling [30–35]. So far, only a few successful examples of fluorescence enhancement sensors for Cr^3+^ have been reported [36–40]. Thus, developing new and effective fluorescence turn-on chemosensors for Cr^3+^ is necessary.

In the process of our research, a tetradentate compound bearing a fluorophore aroused our interest. In this paper, we report the synthesis of a novel anthracene-based tetraperimidine hexafluorophosphate **3**, and its structure was determined by X-ray analysis as well as ^1^H and ^13^C NMR spectroscopy. Particularly, compound **3** was tested as a chemosensor for the recognition of Cr^3+^ through fluorescence, UV, IR, and ^1^H NMR spectroscopy along with HRMS. Altogether, the results indicate the utility of **3** as an effective chemosensor for Cr^3+^.

## Results and Discussion

### Synthesis and characterization of **3**

**Scheme 1:**
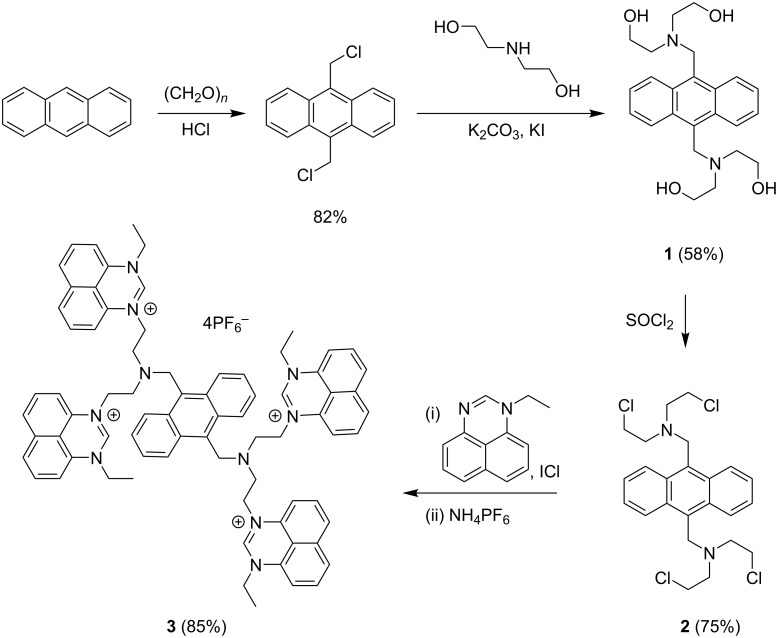
Synthetic route to compound **3**.

As displayed in Scheme 1, paraformaldehyde was reacted with anthracene to give 9,10-di(chloromethyl)anthracene in 82% yield, which reacted further with HN(CH_2_CH_2_OH)_2_ to form 9,10-bis{[*N*,*N*-di(2-hydroxyethyl)amino]methyl}anthracene (**1**) with a yield of 58% [41]. Compound **1** was then treated with SOCl_2_ to generate 9,10-bis{[*N*,*N*-di(2-chloroethyl)amino]methyl}anthracene (**2**) in 75% yield, which reacted with 1-ethylperimidine in the presence of KI to afford the analogous iodide salt to tetraperimidine **3**. Subsequently, an anion exchange reaction with NH_4_PF_6_ was performed to generate tetraperimidine hexafluorophosphate **3** with a yield of 85%. Compound **3** was stable to heat, moisture, and air, and it had a good solubility in DCM, DMSO, and CH_3_CN. In turn, it had a poor solubility in benzene and petroleum ether. In the ^1^H NMR spectrum of **3**, the proton signal corresponding to the NC*H*N motif in perimidine was present at δ = 8.69 ppm [42].

### Structure elucidation of compound **3**

**Figure 1:**
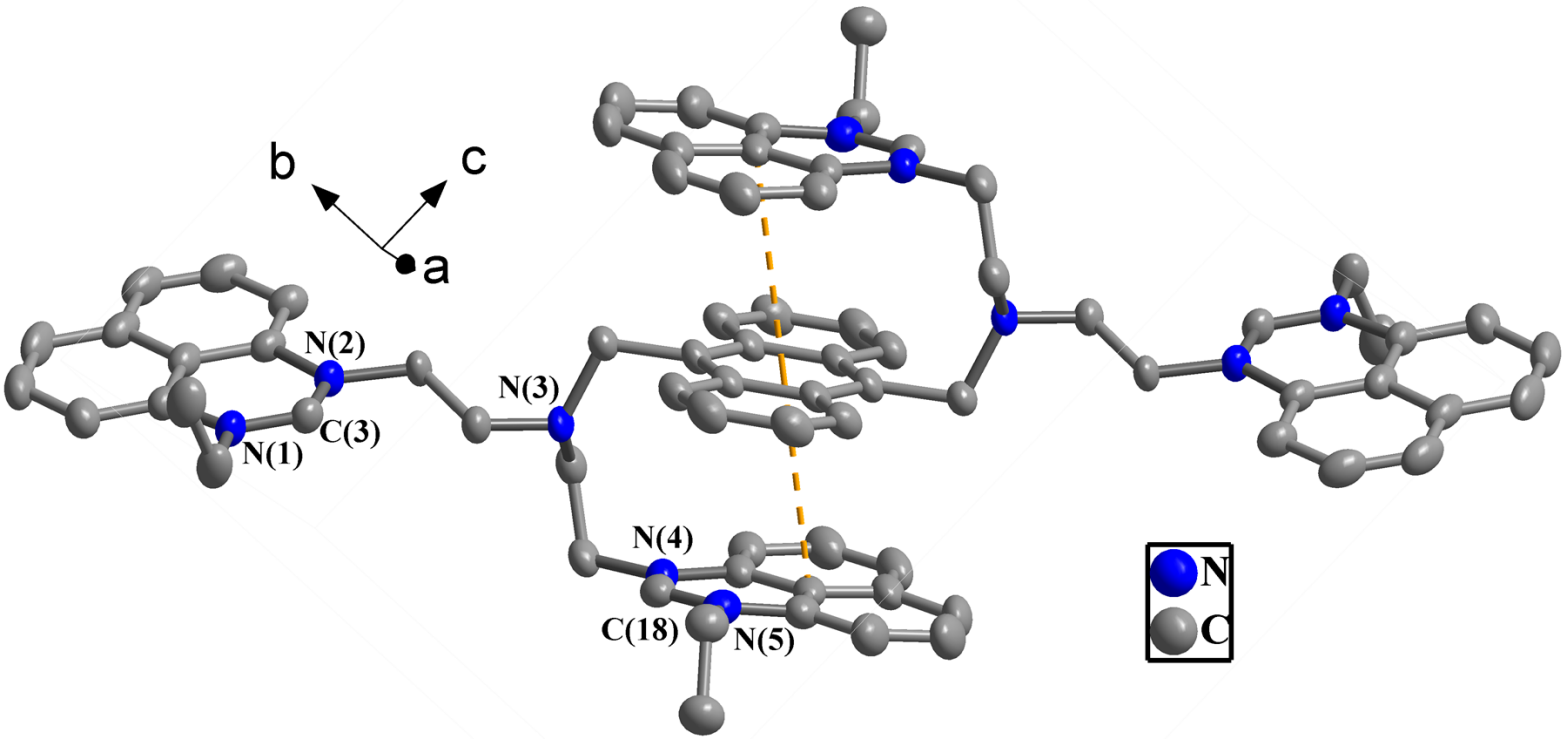
View of the molecular structure of the cationic moiety of **3** in the crystal. Selected bond angles and lengths are N(2)–C(3)–N(1): 125.2(3)°; N(5)–C(18)–N(4): 124.3(4)°; C(3)–N(1): 1.310(5) Å; and C(3)–N(2): 1.315(5) Å.

As can be seen in the crystal structure of **3** in Figure 1, the cationic moiety of the complex contained two (*N*-ethylperimidinyl–C_2_H_4_)_2_NCH_2_– arms attached to the 9- and 10-positions of anthracene, and the dihedral angle between two perimidine units of each arm was determined to be 18.1(4)°. Two of the four perimidine groups were parallel to the anthracene plane, with intramolecular π–π interactions [43] being present in this setup (with a face-to-face distance of 3.566(1) Å between perimidine and anthracene and a center-to-center distance of 3.664(4) Å). The bond distances C(3)–N(1) and C(3)–N(2) were 1.310(5) and 1.315(5) Å and the dihedral angles N(2)–C(3)–N(1) and N(4)–C(18)–N(5) were 125.2(3) and 124.3(4)° [42]. Further, a 1D polymeric chain of **3** monomers was generated through intermolecular π–π interactions between perimidine moieties (with a face-to-face distance of 3.558(4) Å and a center-to-center distance of 3.566(1) Å), as shown in Supporting Information File 1, Figure S1a. Besides, a 2D supramolecular layer was formed by the 1D supramolecular chains through two types of C–H···F hydrogen bonds, namely C(3)–H(3A)···F(2) and C(17)–H(17A)···F(2) interactions (Supporting Information File 1, Figure S1b).

### Chemosensing of cations by **3**

**Figure 2:**
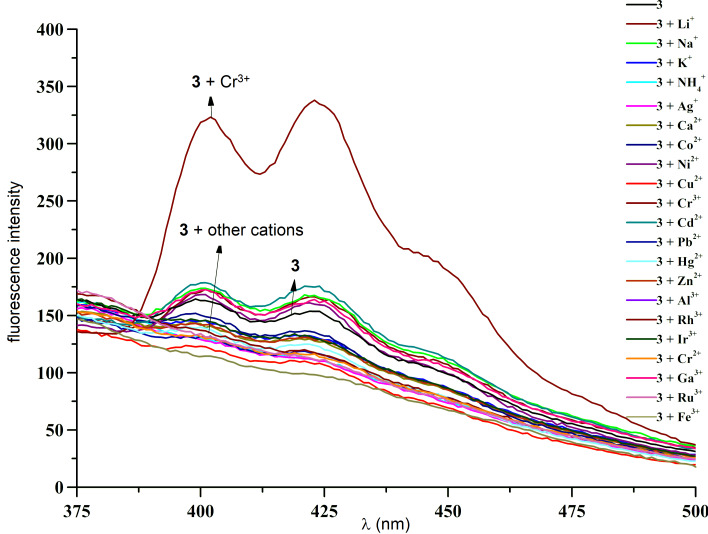
Fluorescence spectra of **3** (*c* = 5.0⋅10^−6^ M) upon addition of 30 equiv of salts of K^+^, Na^+^, Li^+^, Ag^+^, NH_4_^+^, Zn^2+^, Cd^2+^, Ca^2+^, Ni^2+^, Pb^2+^, Cu^2+^, Co^2+^, Al^3+^, Cr^3+^, Hg^+^, Hg^2+^, Rh^3+^, Ir^3+^, Cr^2+^, Ga^3+^, Ru^3+^, and Fe^3+^, respectively (*c* = 1.5⋅10^−4^ M) in CH_3_CN/DMSO, 9:1, v/v at room temperature (excitation wavelength λ_ex_ = 258 nm).

Compound **3** was employed as a host to study its ability to detect some cations through fluorescence and UV titrations in CH_3_CN/DMSO, 9:1, v/v at room temperature. In its free form, three emission bands at 402, 423, and 447 nm were observed at *c* = 5.0⋅10^−6^ M, which were ascribed to the emission of anthracene (Figure 2). When adding 30 equiv of K^+^, Na^+^, Li^+^, Ag^+^, NH_4_^+^, Zn^2+^, Cd^2+^, Ca^2+^, Ni^2+^, Pb^2+^, Cu^2+^, Co^2+^, Al^3+^, Hg^+^, Hg^2+^, Rh^3+^, Ir^3+^, Cr^2+^, Ga^3+^, Ru^3+^, and Fe^3+^, respectively, the intensities of the emission bands did not change significantly. However, a strong enhancement of the emission intensity in the region of 388–500 nm was observed after the addition of 30 equiv of Cr^3+^. Moreover, the absorption peak of **3** at 258 nm (ε = 3.5⋅10^3^ mol^−1^⋅dm^3^⋅cm^−1^) did not exhibit any remarkable response to the addition of these cations, except for Cr^3+^ (ε = 1.1⋅10^4^ mol^−1^⋅dm^3^⋅cm^−1^) as a result of **3**·Cr^3+^ formation (Supporting Information File 1, Figure S2). These results show that **3** is able to effectively distinguish Cr^3+^ from other cations.

**Figure 3:**
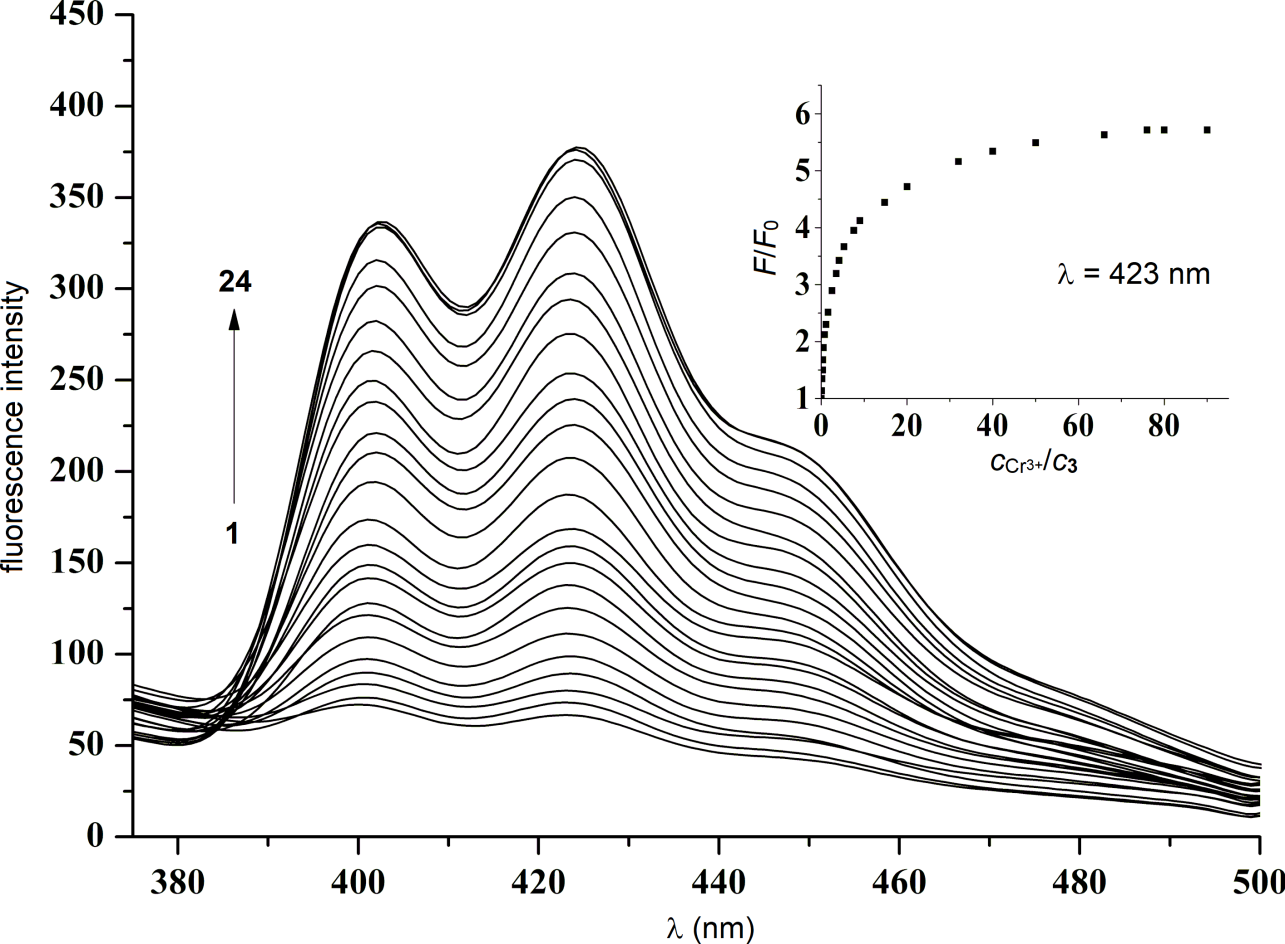
Fluorescence spectra of **3** (*c* = 5⋅10^−6^ M) upon addition of various amounts of Cr^3+^. c_Cr_^3+^ for curves 1–24 (from bottom to top) were (0.00, 0.04, 0.08, 0.16, 0.24, 0.35, 0.49, 0.67, 0.80, 1.00, 1.45, 1.85, 2.30, 3.70, 4.50, 7.00, 10.00, 16.00, 20.00, 25.00, 33.00, 38.00, 40.00, and 45.00)⋅10^−5^ M (λ_ex_ = 258 nm).

To further investigate the recognition of Cr^3+^ by **3**, fluorescence titrations were carried out (Figure 3). The fluorescence intensity of **3** in the region of 388–500 nm increased gradually upon addition of Cr^3+^ (*c* = 5.0⋅10^−6^ M). Titration was continued until no more notable changes in emission intensity occurred. In the inset of Figure 3, it is shown that when the molar ratio of Cr^3+^ to **3**, i.e., *c*_Cr_3+/*c***_3_**, was below 1:1, fluorescence intensity enhanced sharply. However, when the molar ratio exceeded 1:1, the rate of fluorescence enhancement gradually slowed down until no more changes were noticeable. The limit of detection (LOD) was calculated to be 2.33⋅10^−7^ M (Supporting Information File 1, Figure S3). This value is analogous to the lowest corresponding value that has been reported in the literature (9.40⋅10^−7^–5.55⋅10^−6^ M) [44–46]. The association constant *K*_SV_ was calculated to be 6.6⋅10^4^ M^−1^ (*R* = 0.998) for **3**·Cr^3+^ using Equation 1 (Supporting Information File 1, Figure S4) [47].

[1] $${F/F}_{0}=\;1\;+K_{\text{SV}}\cdot c_{\text{Cr}}{}_{{}^{\text{3+}}}$$

In Equation 1, the fluorescence intensities of **3** in the presence of Cr^3+^ and in its free form are represented by *F* and *F*_0_.

In the UV titration experiments, the absorption band in the region of 240–265 nm increased gradually upon addition of Cr^3+^ to a solution of **3** (*c* = 5.0⋅10^−6^ M) in CH_3_CN/DMSO, 9:1, v/v at room temperature (Supporting Information File 1, Figure S5). To evaluate the stability of **3**·Cr^3+^, the stability constant *K* for the complex was computed as 8.23⋅10^4^ M^−1^ (*R* = 0.999) at 258 nm using Equation 2 (Supporting Information File 1, Figure S6) [48–51].

[2] $$A_{0}/\left(A_{0}-A\right)\;=\;[\varepsilon_{\text{r}}/\left(\varepsilon_{\text{r}}-\varepsilon_{\text{c}}\right)]\cdot (1/Kc_{3}+\;1)\;$$

In Equation 2, the absorbances of **3** in presence and absence of Cr^3+^ are represented as *A*_0_ and *A*. The discrepancy in absorbance in the presence and absence of Cr^3+^ is represented through the expression *A*_0_ − *A* (i.e., Δ*A*). The molar extinction coefficients of Cr^3+^ and the complex **3**·Cr^3+^ are represented by ε_r_ and ε_c_.

The complexation stoichiometry between **3** and Cr^3+^ was established by using Job’s method (inset of Figure S5, Supporting Information File 1). When the molar fraction (χ) of **3** was 0.5, the Δ*A*χ value for **3**·Cr^3+^ reached a maximum, which indicated that the complexation stoichiometry between **3** and Cr^3+^ was 1:1 in **3**·Cr^3+^ [8,52–53].

To measure the selectivity of Cr^3+^ complexation by **3**, displacement experiments were carried out (Supporting Information File 1, Figure S7). Firstly, 30 equiv of Cr^3+^ were added to solutions of **3** containing 30 equiv of K^+^, Na^+^, Li^+^, Ag^+^, NH_4_^+^, Zn^2+^, Cd^2+^, Ca^2+^, Ni^2+^, Pb^2+^, Cu^2+^, Co^2+^, Al^3+^, Hg^+^, Hg^2+^, Rh^3+^, Ir^3+^, Cr^2+^, Ga^3+^, Ru^3+^, and Fe^3+^, respectively. The emission intensities of the resulting mixtures were similar to that of a solution containing only **3** and Cr^3+^. These experimental results show that **3** can capture Cr^3+^ selectively while neglecting other cations, with no remarkable interference being caused in the presence of the latter.

In order to probe whether the anions of the chromium(III) salts had effects on the binding of **3** and Cr^3+^, other chromium(III) salts, CrCl_3_, CrBr_3_, Cr_2_(SO_4_)_3_, Cr(NO_3_)_3_, and Cr(OAc)_3_, were tested. As displayed in Figure S8, Supporting Information File 1, when 30 equiv of any of these were added to **3**, similar fluorescence intensities could be detected. A reversible binding experiment was also carried out (Supporting Information File 1, Figure S9). Therein, 30 equiv of ethylenediaminetetraacetic acid (EDTA) were added to a solution of Cr^3+^ (*c* = 1.5⋅10^−4^ M) and **3** (*c* = 5.0⋅10^−6^ M), which led to a reduction of the fluorescence intensity at 388–500 nm. This reduced fluorescent intensity was analogous to that of free **3**, displaying that **3** was regenerated in its uncomplexed form. When Cr^3+^ was added anew, the fluorescent intensity increased again. These results show that the binding process of **3** and Cr^3+^ has good reversibility and highlights the regenerative capacity of the **3**⋅Cr^3+^ complex.

### Interactions between **3** and Cr^3+^

**Scheme 2:**
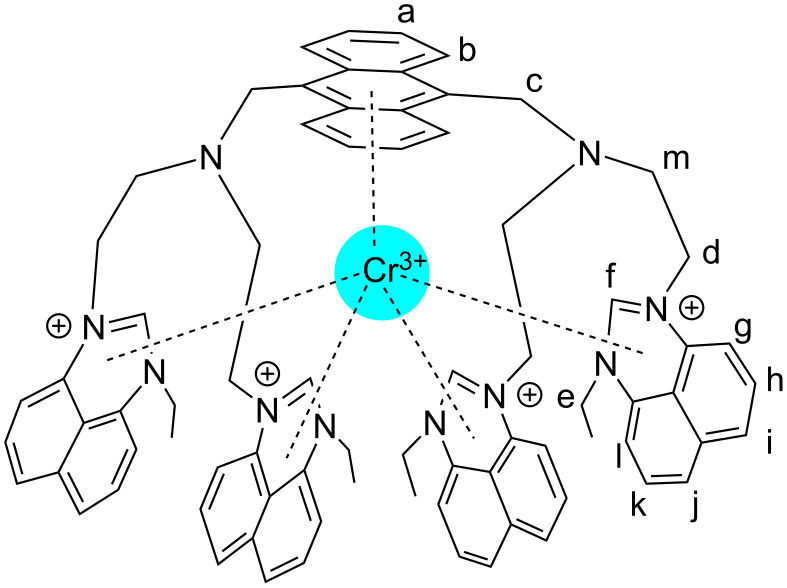
Illustration of interactions between **3** and Cr^3+^ in **3**⋅Cr^3+^.

**Figure 4:**
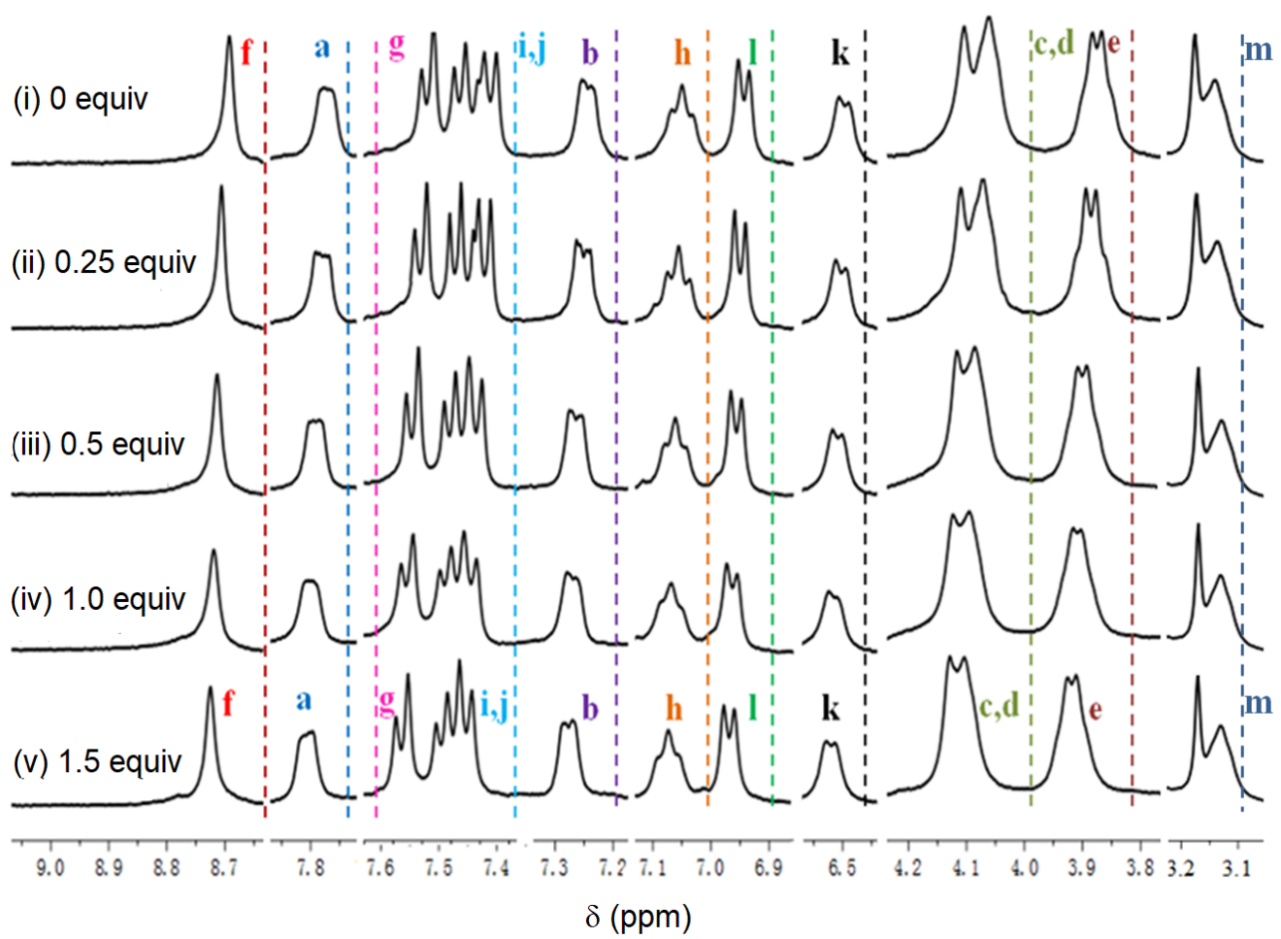
^1^H NMR spectra of **3** in the presence of Cr^3+^ in DMSO-*d*_6_. (i) **3**. (ii) **3** + 0.25 equiv of Cr^3+^. (iii) **3** + 0.5 equiv of Cr^3+^. (iv) **3** + 1 equiv of Cr^3+^. (v) **3** + 1.5 equiv of Cr^3+^.

Looking at the structural characteristics of **3**, the nitrogen atoms of the tertiary amines, and the π systems, were most likely the binding sites for Cr^3+^ through Cr^3+^···N and Cr^3+^···π interactions (Scheme 2). In order to obtain further information on the binding pattern between **3** and Cr^3+^, ^1^H NMR titration studies were done in DMSO-*d*_6_. The spectra are depicted in Figure 4.

Upon incremental addition of Cr^3+^ to **3** (from 0.0 to 1.0 equiv), the proton signals a and b, corresponding to anthracene, shifted downfield by 0.03 ppm in total while the proton signals f and l, corresponding to perimidine, shifted downfield between 0.02 and 0.07 ppm. Further, the proton signals c, d, and e of the CH_2_ groups beside perimidine and anthracence shifted downfield by 0.03 ppm while the chemical shifts of other protons did not undergo visible changes. These experimental results suggest Cr^3+^···π interactions as the most likely binding mode between Cr^3+^ and **3**, as illustrated in Scheme 2. In **3**, each perimidine moiety is electron-rich due to the existence of a 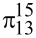 bond (Supporting Information File 1, Figure S10), and the strong affinity of the π system of each perimidine and the anthracene motif towards Cr^3+^ resulted in the facile formation of Cr^3+^···π interactions. It is worth noting that Cr^3+^···π interactions are not uncommon, and they have been reported in diaryl chromium complexes [54–55]. Besides, Cr^3+^···N interactions in **3**·Cr^3+^ did not appear relevant for complexation. The reasons were that (1) the signal m, corresponding to the CH_2_ fragment beside the nitrogen atom of the tertiary amine, did not shift discernibly during ^1^H NMR titration; (2) if the tertiary amine groups coordinated to one Cr^3+^ ion each, a 1:2 binding mode would have been determined for **3**⋅Cr^3+^; and (3) from the molecular structure of **3**, due to the distance, it is spatially impossible that one Cr^3+^ ion is bound by both tertiary amine functions at the same time. All these arguments underline the absence of Cr^3+^···N interactions. The selectivity of the Cr^3+^ binding process by **3** may be mainly due to the metal ion's size, which could have been particularly suitable for coordination between anthracence and four perimidine groups, whereas the sizes of other metal cations were unsuitable. The fact that chromium(III) is triply charged may not have been a key influence because otherwise, other metal cations M^3+^ would have also been bound by **3**.

Furthermore, looking at Figure 4, the proton signals a to l remained unchanged after 1 equiv of Cr^3+^ had been added to **3**. That is, the signals in spectra (iv) and (v) have the same positions. This again illustrates 1:1 complexation between **3** and Cr^3+^. This result is consistent with the conclusions obtained from Job’s plot. In addition, HRMS analysis of **3**·Cr^3+^ (Supporting Information File 1, Figure S11) produced a distinctive signal at *m*/*z* = 587.1086, matching (**3**⋅Cr^3+^)/3, again indicating 1:1 complexation. In the IR spectra of **3** and **3**⋅Cr^3+^ (Supporting Information File 1, Figure S12), the C–C absorption band of a benzene moiety in **3** shifted from 1170 to 1185 cm^−1^ upon complexation, and the C=N absorption band at 1664 cm^−1^ shifted to a value of 1672 cm^−1^.

## Conclusion

In summary, a new anthracene-based tetraperimidine hexafluorophosphate **3** was prepared, and its structure was determined through X-ray analysis and ^1^H and ^13^C NMR spectroscopy. Compound **3** was proved to be a highly sensitive and selective chemosensor for Cr^3+^, and it can effectively distinguish Cr^3+^ from other cations through fluorescence enhancement. Thus, complex **3** may have potential value for the application as a Cr^3+^ detector.

## Experimental

### Materials and instruments

The solvents and chemicals used for synthesis and analysis were analytical grade and obtained commercially. A RF-5301PC ﬂuorescence spectrophotometer (Shimadzu) was used to record fluorescence spectra at room temperature. The excitation and emission slits were set to 10 nm. UV–vis absorption spectra were recorded at room temperature using a JASCO-V570 spectrometer. A Varian spectrometer was employed to record ^1^H and ^13^C NMR spectra. A Perkin-Elmer 2400C Elemental Analyzer was employed for elemental analyses. IR spectra were measured with a PerkinElmer Spectrum 100 FT-IR spectrophotometer. A Q-TOF LC/MS (Agilent) and a VG ZAB-HS (VG) mass spectrometer were applied for HRMS analysis. Melting points were recorded employing a Boetius Block apparatus.

### Analytical data

**Synthesis of 1-ethylperimidine:** Through a dropping funnel, a solution of perimidine (1.43 g, 8.5 mmol) in dry THF (30 mL) was added to a suspension of NaH (0.479 g, 20 mmol) in dry THF (10 mL), followed by stirring at room temperature for 1 h. Subsequently, bromoethane (1.308 g, 12 mmol) was added to the suspension, and the mixture was reacted for 24 h at ambient temperature. After filtration, the solvent was removed at reduced pressure, and water (50 mL) was added to the residue. This was extracted with CHCl_3_ (3 × 20 mL), the organic layer was rinsed with water (3 × 30 mL), and dried over anhydrous MgSO_4_. 1-Ethylperimidine was obtained as a yellow-green solid (0.825 g, 49%) after removal of the solvent. mp 198–200 °C; ^1^H NMR (400 MHz, DMSO-*d*_6_) δ 1.49 (t, *J* = 7.2 Hz, 3H, C*H*_3_), 4.27 (q, *J* = 7.2 Hz, 2H, C*H*_2_), 7.31 (d, *J* = 8.8 Hz, 2H, Ar*H*), 7.76 (d, *J* = 8.8 Hz, 2H, Ar*H*), 8.04 (s, 1H, Ar*H*), 8.25 (s, 1H, Ar*H*), 9.73 ppm (s, 1H, NC*H*N).

**Synthesis of 9,10-bis(chloromethyl)anthracene**: A suspension of paraformaldehyde (6.155 g, 205 mmol) and anthracene (17.823 g, 100 mmol) in a mixture of acetic acid (50 mL) and hydrochloric acid (36%, 20 mL) was heated to 100 °C for 5 h. Then, 200 mL of water were added to precipitate a yellow solid. After filtration, 9,10-bis(chloromethyl)anthracene was obtained as a yellow powder (22.452 g, 82%). mp 246–248 °C; ^1^H NMR (400 MHz, CDCl_3_) δ 5.62 (s, 4H, C*H*_2_), 7.68 (q, *J* = 1.6 Hz, 4H, Ar*H*), 8.41 ppm (q, *J* = 1.6 Hz, 4H, Ar*H*); ^13^C NMR (100 MHz, CDCl_3_) δ 130.2 (Ar*C*), 129.7 (Ar*C*), 126.7 (Ar*C*), 124.3 (Ar*C*), 67.1 ppm (*C*H_2_).

**Synthesis of 9,10-bis{[*N*,*N*-di(2-hydroxyethyl)amino]methyl}anthracene (1):** A suspension of diethanolamine (9.988 g, 95 mmol) and K_2_CO_3_ (25.015 g, 181 mmol) in 100 mL of CH_3_CN/CHCl_3_, 1:1, v/v was stirred under reflux for 1 h. Subsequently, 9,10-bis(chloromethyl)anthracene (8.255 g, 30 mmol) and KI (0.914 g, 5.5 mmol) were added to the solution, and the mixture was reacted for 30 h at 35 °C. Then, the solvent was evaporated in vacuo to give a yellow oil. After rinsing with water, a yellow solid, 9,10-bis{[*N*,*N*-di(2-hydroxyethyl)amino]methyl}anthracene (**1**), was obtained (7.189 g, 58%). mp 153–155 °C; ^1^H NMR (400 MHz, CDCl_3_) δ 1.96 (s, 4H, O*H*), 2.77 (t, *J* = 5.3 Hz, 8H, C*H*_2_), 3.43 (t, *J* = 5.3 Hz, 8H, C*H*_2_), 4.74 (s, 4H, C*H*_2_), 7.55 (q, *J* = 3.3 Hz, 4H, Ar*H*), 8.51 ppm (q, *J* = 3.3 Hz, 4H, Ar*H*); ^13^C NMR (100 MHz, CDCl_3_) δ 130.9 (Ar*C*), 130.7 (Ar*C*), 125.8 (Ar*C*), 125.1 (Ar*C*), 59.8 (*C*H_2_), 56.1 (*C*H_2_), 51.6 ppm (*C*H_2_).

**Synthesis of 9,10-bis{[*N*,*N*-di(2-chloroethyl)amino]methyl}anthracene (2):** A solution of SOCl_2_ (9.517 g, 80 mmol) in dioxane (30 mL) was added dropwise to a solution of compound **1** (4.125 g, 10 mmol) in dioxane (50 mL). The reaction mixture was stirred at 30 °C for 3 days, during which the formation of a yellow precipitate occurred. After filtration, the hydrochloride of 9,10-bis{[*N*,*N*-di(2-chloroethyl)amino]methyl}anthracene (**2**) was obtained as a yellow solid. This was rinsed with 200 mL of a NaOH solution (20%) for neutralization and extracted with CHCl_3_ (3 × 60 mL). The organic layer was washed with water (3 × 20 mL) and dried over anhydrous MgSO_4_. A yellow powder of 9,10-bis{[*N*,*N*-di(2-chloroethyl)amino]methyl}anthracene (**2**) was obtained (3.67 g, 75%) after removal of CHCl_3_. mp 131–133 °C; ^1^H NMR (400 MHz, DMSO-*d*_6_) δ 2.92 (t, *J* = 6.7 Hz, 8H, C*H*_2_), 3.52 (q, *J* = 6.1 Hz, 8H, C*H*_2_), 4.74 (s, 4H, C*H*_2_), 7.55 (q, *J* =3.4 Hz, 4H, Ar*H*), 8.60 ppm (q, *J* = 3.4 Hz, 4H, Ar*H*); ^13^C NMR (100 MHz, DMSO-*d*_6_) δ 130.9 (Ar*C*), 130.8 (Ar*C*), 126.0 (Ar*C*), 125.8 (Ar*C*), 55.4 (*C*H_2_), 50.3 (*C*H_2_), 42.7 ppm (*C*H_2_).

**Synthesis of 3,3',3'',3'''-(((anthracene-9,10-diylbis(methylene))bis(azanetriyl))tetrakis(ethane-2,1-diyl))tetrakis(1-ethyl-1*H*-perimidin-3-ium) tetra(hexafluorophosphate) (3):** A mixture of 1-ethylperimidine (1.177 g, 6 mmol), 9,10-bis{[*N*,*N*-di(2-chloroethyl)amino]methyl}anthracene (**2**, 0.389 g, 0.8 mmol), and KI (0.5 g, 3 mmol) in 30 mL of DMF/dioxane, 1:4, v/v was stirred under reflux for 5 days. After the solvent was removed, water (50 mL) was added to the residue. This was extracted with CHCl_3_ (3 × 30 mL), the organic layer was washed with water (3 × 20 mL), and dried over anhydrous MgSO_4_. A yellow powder of the tetraperimidine iodide species was gained after removal of CHCl_3_.

A solution of NH_4_PF_6_ (0.978 g, 6 mmol) and the tetraperimidine iodide compound (1.309 g, 0.8 mmol) in MeOH (100 mL) was stirred at room temperature for 3 days to give a yellow precipitate. This precipitate was collected through filtration and washed with methanol (2⋅10 mL) to afford tetraperimidine hexafluorophosphate **3** (1.158 g, 85%). mp 220–222 °C; anal calcd for C_76_H_76_N_10_P_4_F_24_, C, 53.40; H, 4.48; N, 8.19%; found, C, 53.53; H, 4.26; N, 8.21%; HRESIMS (*m*/*z*): [M − 2PF_6_^−^]^2+^/2 calcd for C_38_H_38_F_6_N_5_P, 709.2769; found, 709.3631; ^1^H NMR (400 MHz, DMSO-*d*_6_) δ 1.37 (s, 12H, C*H*_3_), 3.18 (s, 8H, C*H*_2_), 3.87 (d, *J* = 6.4 Hz, 8H, C*H*_2_), 4.08 (t, *J* = 3.2 Hz, 12H, C*H*_2_), 6.50 (q, *J* = 2.0 Hz, 4H, Ar*H*), 6.94 (d, *J* = 4.4 Hz, 4H, Ar*H*), 7.05 (s, 4H, Ar*H*), 7.23 (m, 4H, Ar*H*), 7.40 (t, *J* = 8.4 Hz, 8H, Ar*H*), 7.45 (s, 4H, Ar*H*), 7.51 (s, 4H, Ar*H*), 8.69 ppm (s, 4H, NC*H*N); ^13^C NMR (100 MHz, DMSO-*d*_6_) δ 152.2 (N*C*N), 134.1 (Ar*C*), 130.7 (Ar*C*), 130.6 (Ar*C*), 129.1 (Ar*C*), 128.1 (Ar*C*), 127.6 (Ar*C*), 124.8 (Ar*C*), 124.4 (Ar*C*), 123.4 (Ar*C*), 120.5 (Ar*C*), 107.9 (Ar*C*), 106.8 (Ar*C*), 48.5 (*C*H_2_), 46.7 (*C*H_2_), 46.6 (*C*H_2_), 38.9 (*C*H2), 11.9 ppm (*C*H_2_).

### Fluorescence titrations

The concentration of **3** and the guest ions was 5.0⋅10^−6^ and 0.0–45.0⋅10^−5^ M, respectively, in the sample solutions. The excitation wavelength was set to λ_ex_ = 258 nm and the widths of the emission and excitation spectral lines were adjusted to 5 and 10 nm. The emission spectra were recorded in the range of 375–500 nm. The program Origin 8.0 was employed for data processing.

### UV–vis titrations

For UV–vis titrations, the sample solutions were prepared analogously to fluorescence titrations. The concentration of **3** was adjusted to 1.0⋅10^−6^ M while the concentration of Cr^3+^ ranged between 0.0 and 36.0⋅10^−6^ M. The absorption spectra were recorded at 240–300 nm. Origin 8.0 was employed for data processing.

### X-ray analysis

Diffraction data of **3** were collected by a Bruker Apex II CCD diffractometer [56]. SHELXS was used to solve the structure of **3** [57]. Other crystallographic data are shown in Supporting Information File 1, Table S1.

## Supporting Information

File 1 Supporting crystallographic data, fluorescence, UV, HRMS, and IR spectra of **3** and **3**·Cr^3+^, general considerations, characterization data, and copies of the ^1^H and ^13^C NMR spectra of all compounds.
