# Supplementary material for: Preparation of anthracene-based tetraperimidine hexafluorophosphate and selective recognition of chromium(III) ions
Source: Beilstein J Org Chem. 2019 Nov 25;15:2847–55. doi: 10.3762/bjoc.15.278 (PMC6902876; doi:10.3762/bjoc.15.278)
Supplement: File 1 — Supporting crystallographic data, fluorescence, UV, HRMS, and IR spectra of 3 and 3·Cr3+, general considerations, characterization data, and copies of the 1H and 13C NMR spectra of all compounds. [file Beilstein_J_Org_Chem-15-2847-s001.pdf]

BEILSTEIN JOURNAL OF ORGANIC CHEMISTRY

Supporting Information

for

Preparation of anthracene-based tetraperimidine hexafluorophosphate and selective recognition of chromium(III) ions

Qing-Xiang Liu, Feng Yang, Zhi-Xiang Zhao, Shao-Cong Yu and Yue Ding

Beilstein J. Org. Chem. **2019,** *15,* 2847–2855. doi:10.3762/bjoc.15.278

Supporting crystallographic data, fluorescence, UV, HRMS, and IR spectra of 3 and 3·Cr^{3+}, general considerations, characterization data, and copies of the ^{1}H and ^{13}C NMR spectra of all compounds

License and Terms: This is a supporting information file under the terms of the Creative Commons Attribution License (http://creativecommons.org/licenses/by/4.0). Please note that the reuse, redistribution and reproduction in particular requires that the authors and source are credited.
The license is subject to the *Beilstein Journal of Organic Chemistry* terms and conditions: (https://www.beilstein-journals.org/bjoc)

Table of contents

1. CCDC numbers for compound **3**.
2. Crystal data and structure refinements for **3** (Table S1).
3. Crystal packing of compound **3** (Figure S1(a) and Figure S1(b).
4. Fluorescence and UV–vis spectroscopy for compound **3** (Figures S2–S9).
5. Figure S10 π_{13}^{15} bond of perimidine.
6. Figure of HRMS for **3**·Cr^{3+} (Figure S11).
7. Infrared spectra of **3** and **3**·Cr^{3+} (Figure S12).
8. Figure of HRMS for **3** (Figure S13).
9. ^{1}H NMR and ^{13}C NMR spectra of intermediate and compound **3** (Figures S14–S22).

1. CCDC number for compound 3.

CCDC 1918752 contains the supplementary crystallographic data for compound The data can be obtained free of charge via http//www.ccdc.cam.ac.uk/conts/retrieving. html, or from the Cambridge Crystallographic Data Centre, 12 Union Road, Cambridge, CB2 1EZ, UK; fax: (+44) 223-336-033; or e-mail: deposit@ccdc.cam. ac. uk.

2. Crystal data and structure refinements for 3

Table S1: Crystal data and structure refinements for **3**

Chemical formula	$C_{76}H_{76}N_{10}P_4F_{24}$ $\cdot 4CH_3CN$	*F*(000)	968
Formula weight	1875.57	Cryst size, mm	0.25 × 0.15 × 0.14
Cryst syst	Triclinic	θ_{min}, θ_{max}, deg	3.74, 67.06
Space group	$P\bar{1}$	*T* /K	172.9(1)
a/Å	11.850(5)	No. of data collected	14087
b/Å	12.401(6)	No. of unique data	7439
c/Å	14.951(5)	No. of refined params	579
α/deg	92.4(1)	Goodness-of-fit on F^2 [a]	1.048
β/deg	94.3(1)	Final *R* indices[b] [$I > 2\sigma(I)$]	
γ/deg	107.3(4)	R_1	0.0754
V/Å^3	2086.65(1)	wR_2	0.1986
Z	1	*R* indices (all data)	
D_{calcd}, Mg/m^3	1.493	R_1	0.1117
Abs coeff, mm^{-1}	1.797	wR_2	0.2238

[a] $GOF = [\Sigma\omega(F_o^2 - F_c^2)^2 / (n - p)]^{1/2}$, where n is the number of reflection and p is the number of parameters refined. [b] $R_1 = \Sigma(||F_o| - |F_c||)/\Sigma|F_o|$; $wR_2 = [\Sigma[w(F_o^2 - F_c^2)^2] / \Sigma w(F_o^2)^2]^{1/2}$.

3. Crystal packing of compound 3

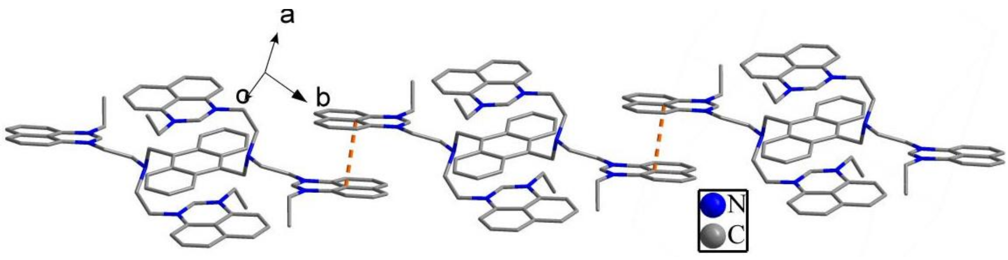

Figure S1(a): 1D supermolecular chain of **3**.

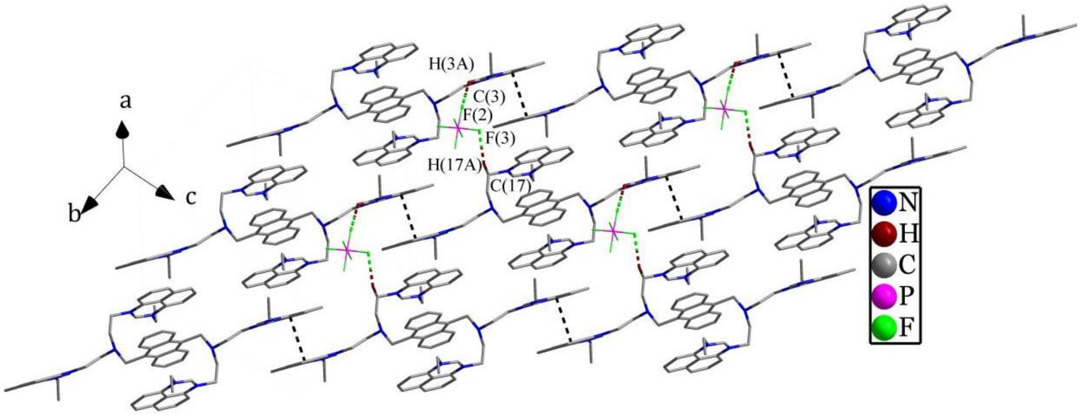

Figure S1(b): 2D supermolecular layer of **3.**

4. Fluorescence and UV–vis spectroscopy for compound 3

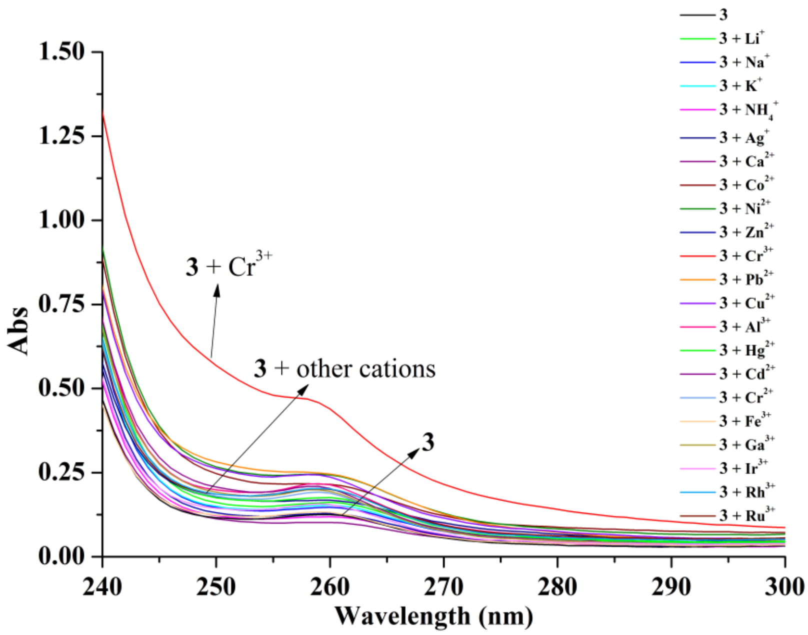

Figure S2: UV–vis absorption change of **3** (5.0×10^{-6} mol/L) upon the addition of the nitrate salts of K^+, Na^+, Li^+, Ag^+, NH_4^+, Zn^{2+}, Cd^{2+}, Ca^{2+}, Ni^{2+}, Pb^{2+}, Cu^{2+}, Co^{2+}, Al^{3+}, Cr^{3+}, Hg^+, Hg^{2+}, Rh^{3+}, Ir^{3+}, Cr^{2+}, Ga^{3+}, Ru^{3+} and Fe^{3+} (5×10^{-5} mol/L) in acetonitrile at 25 °C.

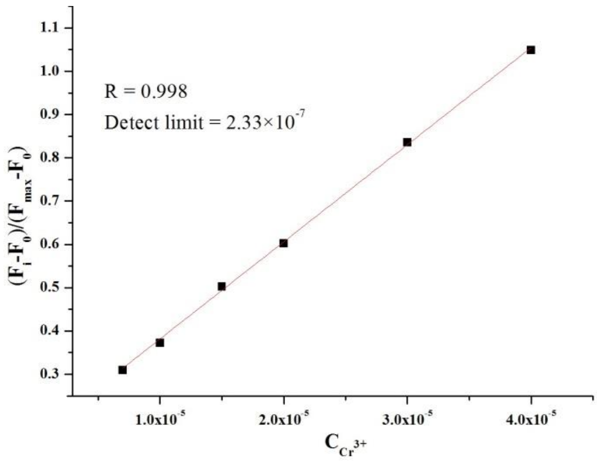

Figure S3: Emission (at 423 nm) of **3** at different concentrations of Cr^{3+} (0.7, 1.0, 1.5, 2.0, 3.0, 4.0 × 10^{-5} mol/L) added, normalized between the minimum emission (0.0 M Cr^{3+}) and the emission 4.0 × 10^{-5} mol/L Cr^{3+}. The detection limit was determined to be 2.33 × 10^{-7} mol/L.

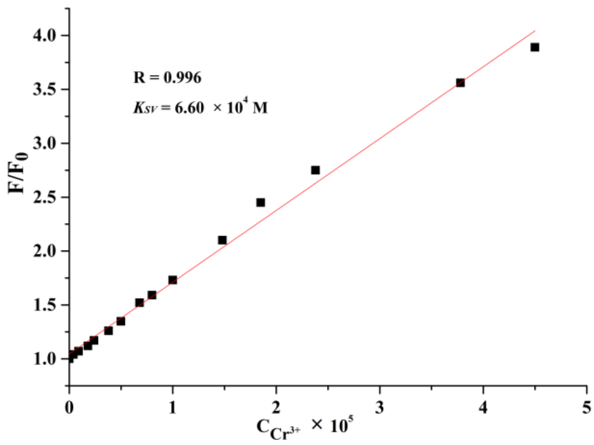

Figure S4: Stern–Volmer plot of **3** (5.0 × 10^{-6} mol/L) in the presence of Cr^{3+} in CH_3CN/DMSO 9:1 (v:v) at 25 °C, and the linear range is from 0.0–4.5 × 10^{-5} mol/L.

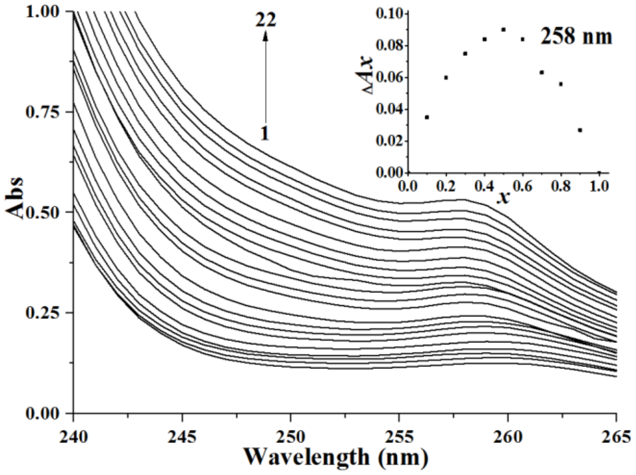

Figure S5: The UV–vis absorption spectra of **3** (5.0 × 10^{-6} mol/L) in CH_3CN/DMSO

9:1 (v/v) at 25 °C. $C_{Cr^{3+}}$ for curves 1–22 are 0, 0.02, 0.06, 0.1, 0.2, 0.4, 0.6, 0.8, 1.2, 1.4, 1.6, 1.8, 2.0, 2.2, 2.4, 2.6, 2.8, 3.0, 3.2, 3.4, 3.5 and 3.6 × 10^{-5}. Inset: the Job's plot for **3**·Cr^{3+} complex in CH_3CN/DMSO 9:1 (v/v) at 258 nm.

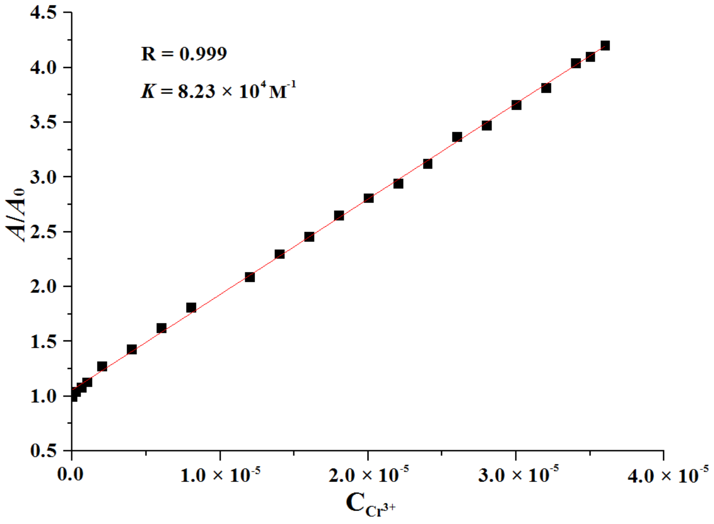

Figure S6: Benesi–Hildebrand plot of **3** (5.0 × 10^{-6} mol/L) at 258 nm in the presence of Cr^{3+} in CH_3CN/DMSO 9:1 (v/v) at 25 °C.

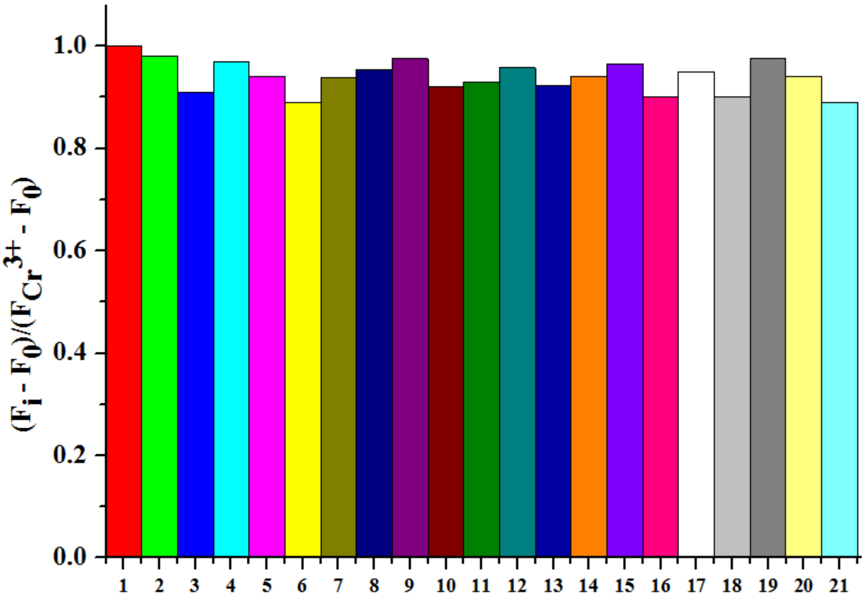

Figure S7: Change ratio $((F_i - F_0)/(F_{Cr^{3+}} - F_0))$ of fluorescence intensity of **3** (5.0 × 10^{-6} mol/L) at 423 nm upon the addition of 30 equiv Cr^{3+} in the presence of 30 equiv background cations. 1: Cr^{3+}; 2: Cr^{3+} + Li^{+}; 3: Cr^{3+} + Na^{+}; 4: Cr^{3+} + K^{+}; 5: Cr^{3+} + NH_4^{+}; 6: Cr^{3+} + Ag^{+}; 7: Cr^{3+} + Ca^{2+}; 8: Cr^{3+} + Co^{2+}; 9: Cr^{3+} + Ni^{2+}; 10: Cr^{3+} + Zn^{2+}; 11: Cr^{3+} + Cu^{2+}; 12: Cr^{3+} + Cd^{2+}; 13: Cr^{3+} + Pb^{2+}; 14: Cr^{3+} + Hg^{2+}; 15: Cr^{3+} + Al^{3+}; 16: Cr^{3+} +

Rh^{3+}; 17: Cr^{3+} + Ir^{3+}; 18: Cr^{3+} + Cr^{2+}; 19: Cr^{3+} + Ga^{3+}; 20: Cr^{3+} + Ru^{3+}; 21: Cr^{3+} + Fe^{3+} in CH_3CN/DMSO 9:1 (v/v) at 25 °C.

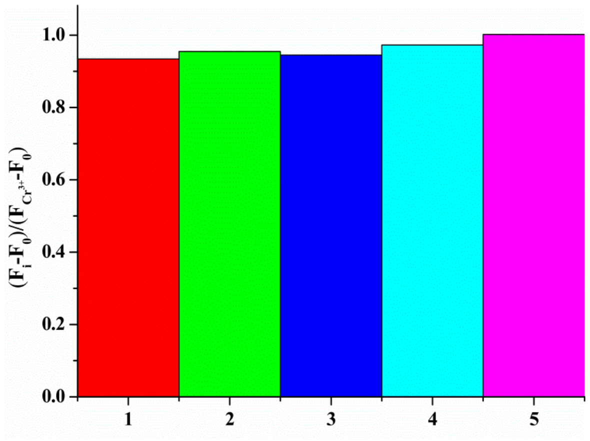

Figure S8: Fluorescence intensity of **3** (5.0×10^{-6} mol/L) in various mixtures of different Cr^{3+} salts (1: $CrCl_3$, 2: $CrBr_3$, 3: $Cr_2(SO_4)_3$, 4: $Cr(OAc)_3$, 5: $Cr(NO_3)_3$; 3.0×10^{-6} mol/L) in CH_3CN/DMSO 9:1 (v/v) at 25 °C.

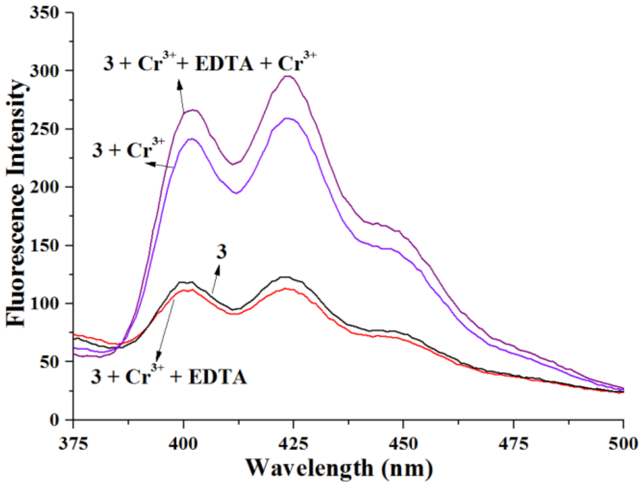

Figure S9: Fluorescence reversibility of **3** upon the detection of Cr^{3+}. Fluorescent changes of **3** after the addition of Cr^{3+}, EDTA, Cr^{3+} in that order in CH_3CN/DMSO 9:1 (v/v) at 25 °C.

5. The Figure S10 π_{13}^{15} bond of perimidine

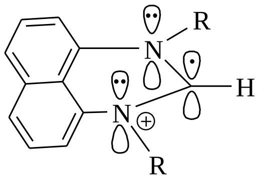

Figure S10: π_{13}^{15} bond of perimidine.

6. Figure of HRMS for 3·Cr^{3+}

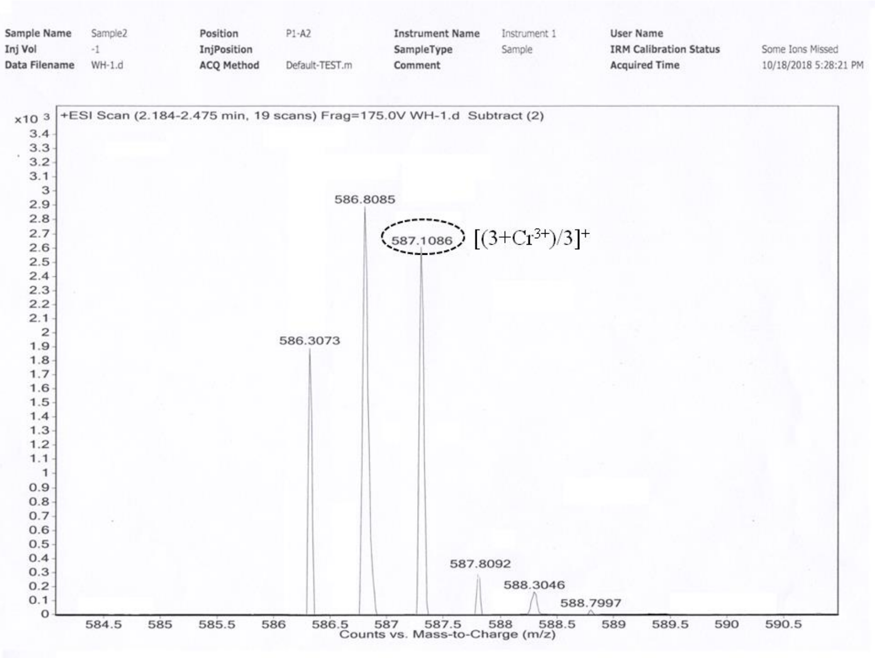

Figure S11: HRMS for **3**·Cr^{3+}.

7. Infrared spectra of 3 and 3·Cr^{3+}

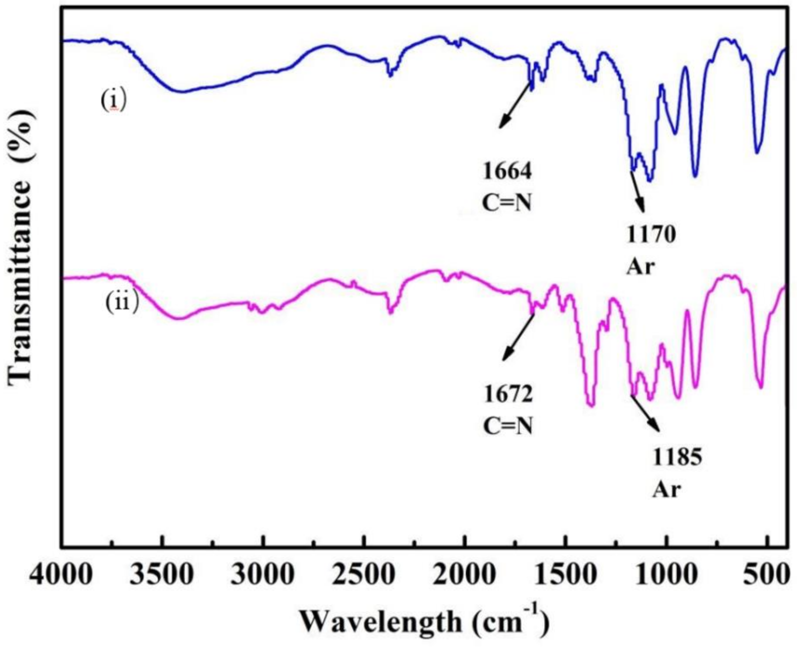

Figure S12: Infrared spectroscopy of **3** (top) and **3·Cr^{3+}** (bottom).

8. Figure of HRMS for 3

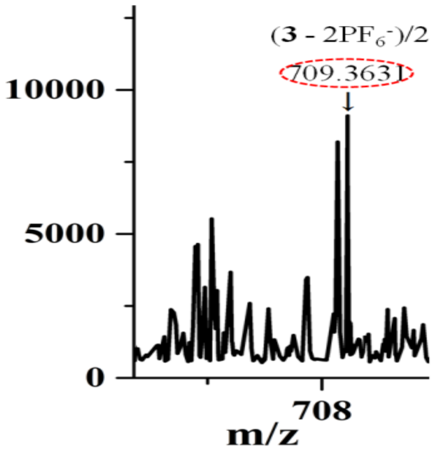

Figure S13: HRMS for **3**.

9. ^{1}H NMR and ^{13}C NMR spectra of intermediate and compound 3

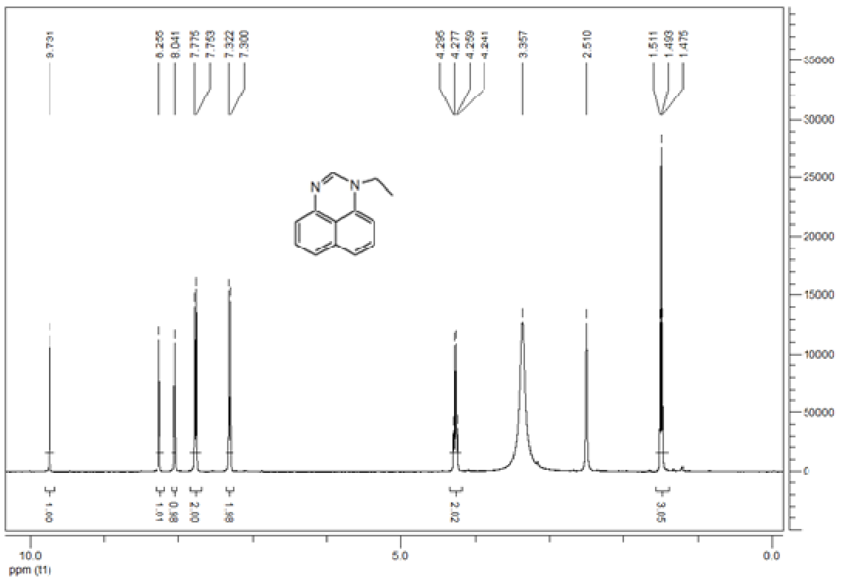

Figure S14: The ^{1}H NMR (400 MHz, DMSO-d_6) spectra of 1-ethylperimidine.

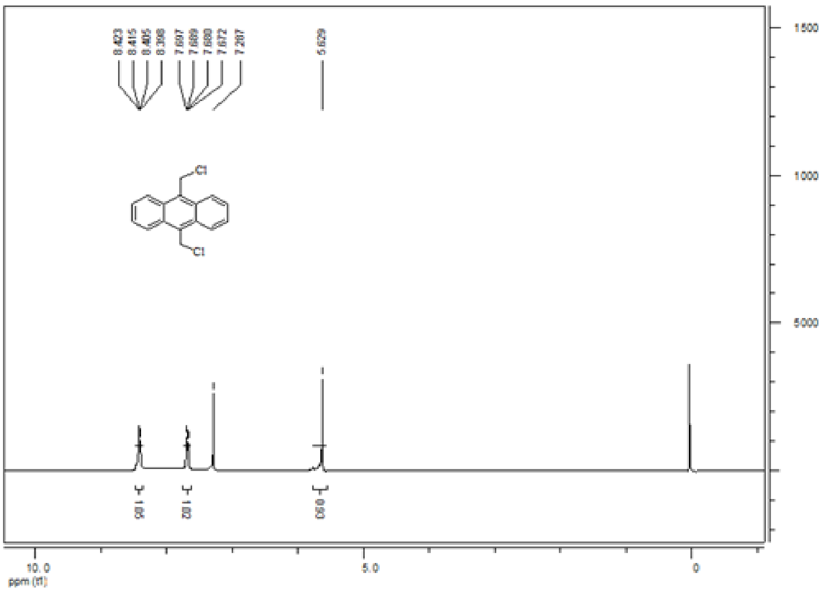

Figure S15: The ^{1}H NMR (400 MHz, $CDCl_3$) spectra of 9,10-bis(chloromethyl)anthracene.

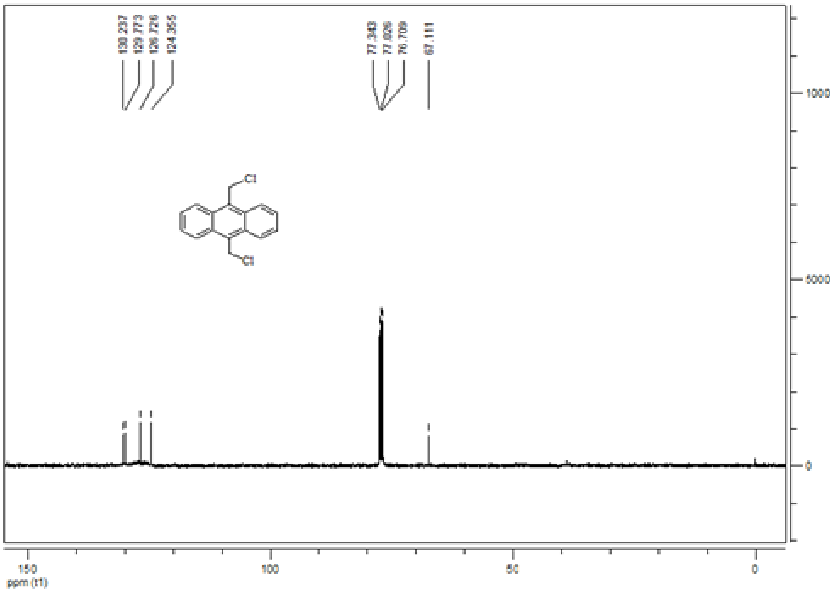

Figure S16: The ^{13}C NMR (100 MHz, $CDCl_3$) spectra of 9,10-bis(chloromethyl)anthracene.

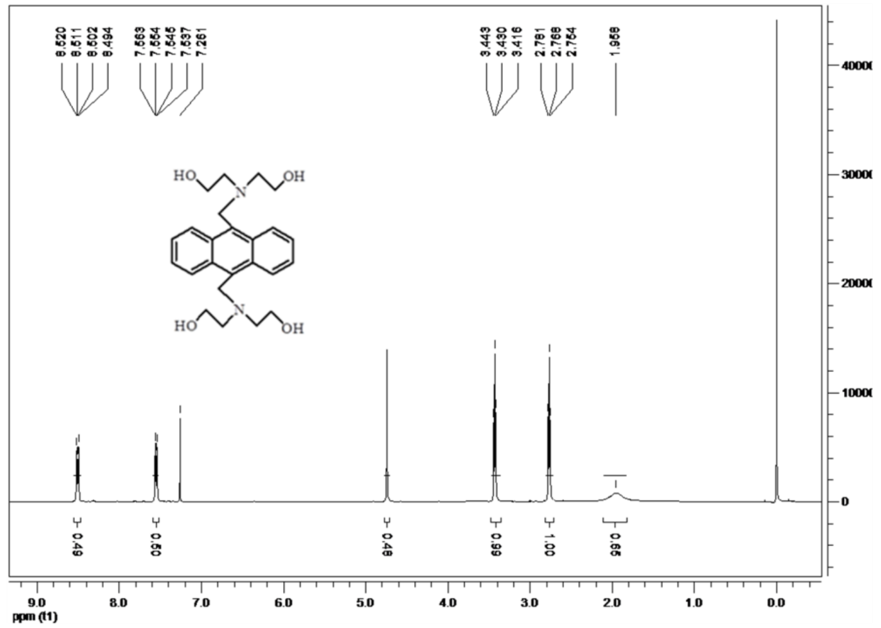

Figure S17: The ^{1}H NMR (600 MHz, $CDCl_3$) spectra of 9,10-bis[{*N*,*N*-di(2-hydroxyethyl)amino}methyl]anthracene.

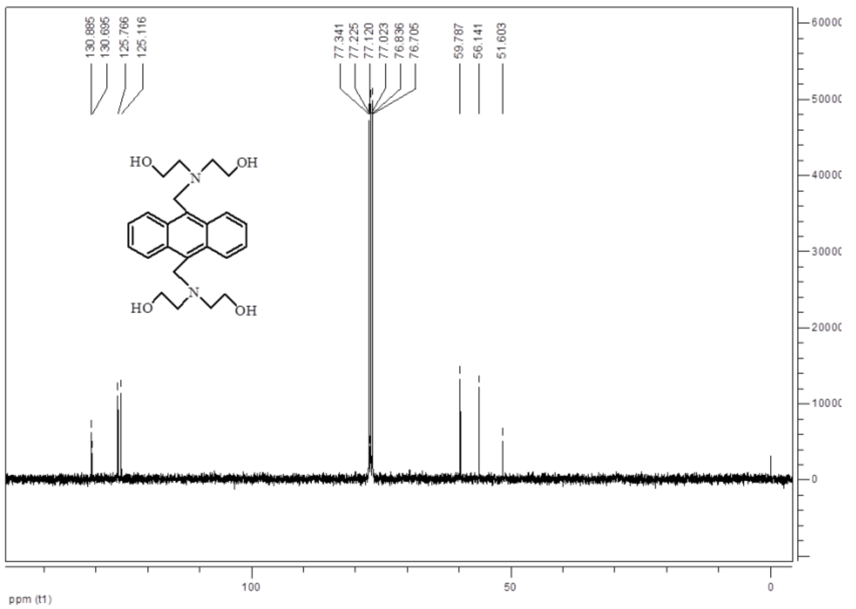

Figure S18: The ^{13}C NMR (150 MHz, $CDCl_3$) spectra of 9,10-bis[{*N*,*N*-di(2-hydroxyethyl)amino}methyl]anthracene.

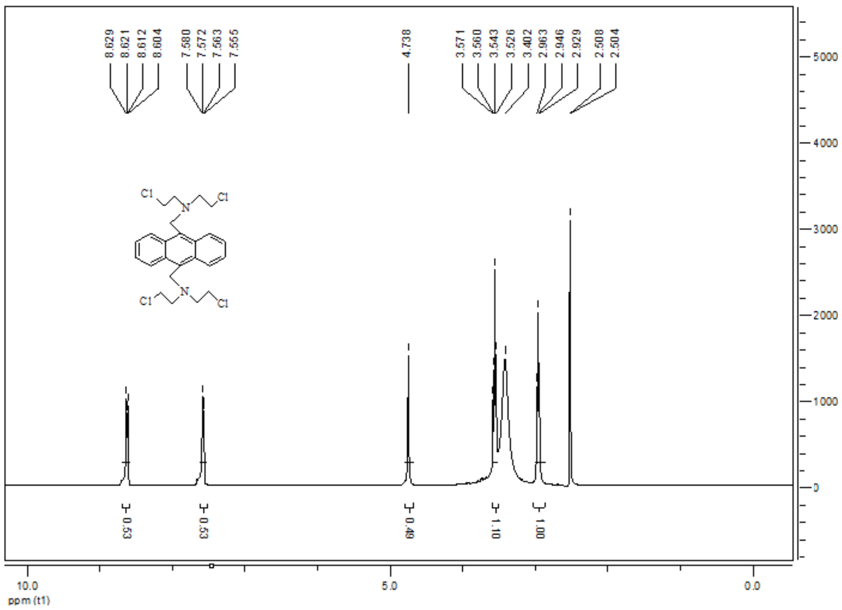

Figure S19: The ^{1}H NMR (600 MHz, DMSO-d_6) spectra of 9,10-bis[{*N*,*N*-di(2-chloroethyl)amino}methyl]anthracene.

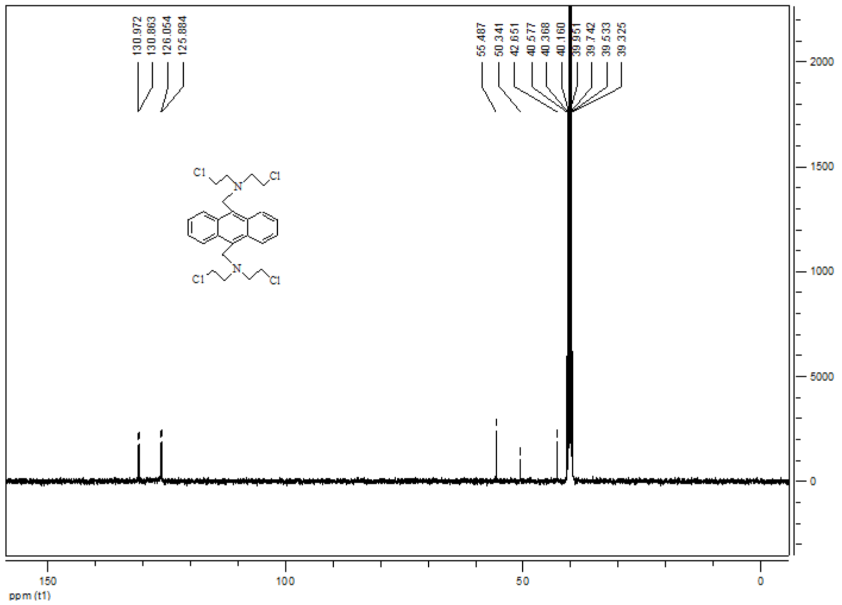

Figure S20: The ^{13}C NMR (150 MHz, DMSO-d_6) spectra of 9,10-bis[{*N*,*N*-di(2-chloroethyl)amino}methyl]anthracene.

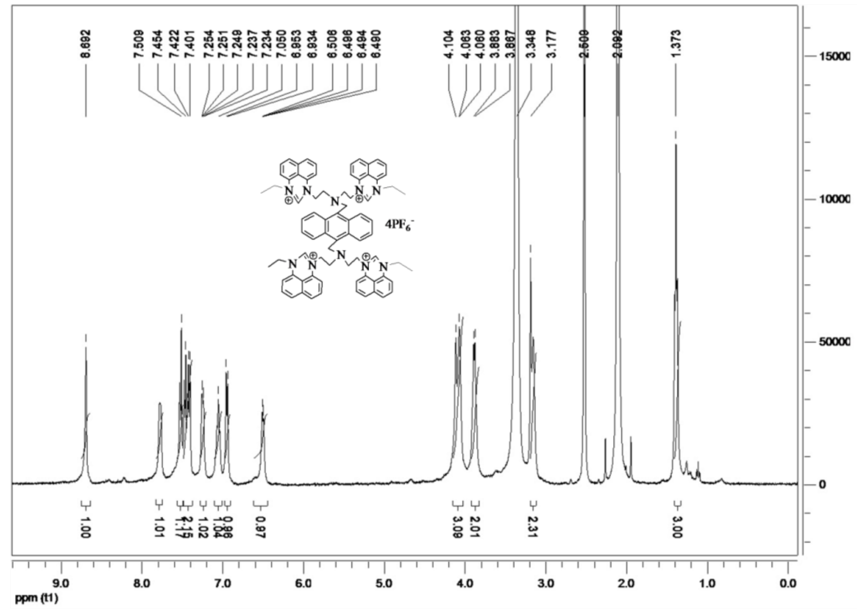

Figure S21: The ^{1}H NMR (600 MHz, DMSO-d_6) spectrum of **3.**

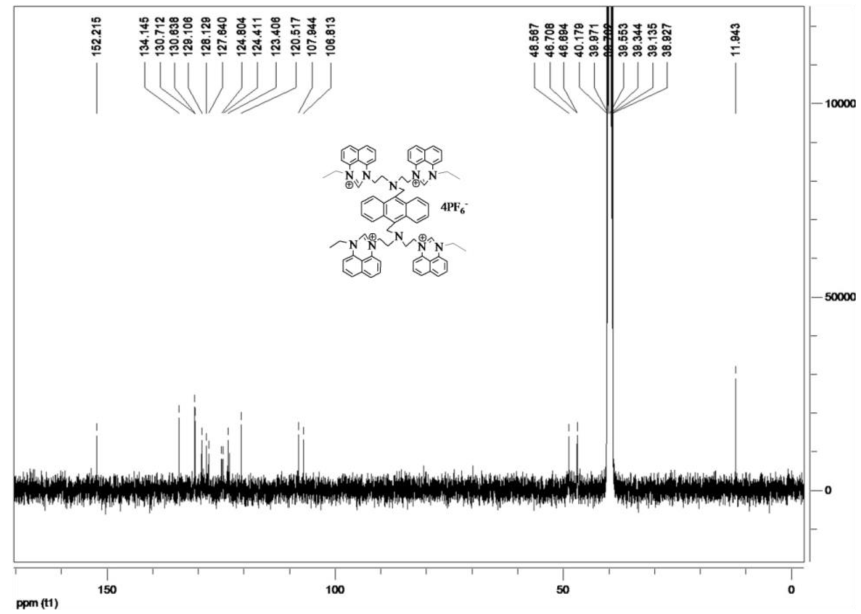

Figure S22: The ^{13}C NMR (150 MHz, DMSO-d_6) spectrum of **3**.
